# Salsalate and Adiponectin Improve Palmitate-Induced Insulin Resistance via Inhibition of Selenoprotein P through the AMPK-FOXO1α Pathway

**DOI:** 10.1371/journal.pone.0066529

**Published:** 2013-06-18

**Authors:** Tae Woo Jung, Hae Yoon Choi, So Young Lee, Ho Cheol Hong, Sae Jeong Yang, Hye Jin Yoo, Byung-Soo Youn, Sei Hyun Baik, Kyung Mook Choi

**Affiliations:** 1 Division of Endocrinology and Metabolism, Department of Internal Medicine, College of Medicine, Korea University, Seoul, Korea; 2 AdipoGen, Inc., Songdo Technopark, Yeonsu-gu, Incheon, Korea; Thomas Jefferson University, United States of America

## Abstract

Selenoprotein P (SeP) was recently identified as a hepatokine that induces insulin resistance (IR) in rodents and humans. Recent clinical trials have shown that salsalate, a prodrug of salicylate, significantly lowers blood glucose levels and increases adiponectin concentrations. We examined the effects of salsalate and full length-adiponectin (fAd) on the expression of SeP under hyperlipidemic conditions and explored their regulatory mechanism on SeP. In palmitate-treated HepG2 cells as well as high fat diet (HFD)-fed male Spraque Dawley (SD) rats and male *db/db* mice, SeP expression and its regulatory pathway, including AMPK-FOXO1α, were evaluated after administration of salsalate and salicylate. Palmitate treatment significantly increased SeP expression and aggravated IR, while knock-down of SeP by siRNA restored these changes in HepG2 cells. Palmitate-induced SeP expression was inhibited by both salsalate and salicylate, which was mediated by AMPK activation, and was blocked by AMPK siRNA or an inhibitor of AMPK. Chromatin immunoprecipitation (ChIP) and electrophoretic mobility shift (EMSA) assay showed that salsalate suppressed SeP expression by AMPK-mediated phosphorylation of FOXO1α. Moreover, fAd also reduced palmitate-induced SeP expression through the activation of AMPK, which results in improved IR. Both salsalate and salicylate treatment significantly improved glucose intolerance and insulin sensitivity, accompanied by reduced SeP mRNA and protein expression in HFD-fed rats and *db/db* mice, respectively. Taken together, we found that salsalate and adiponectin ameliorated palmitate-induced IR in hepatocytes via SeP inhibition through the AMPK-FOXO1α pathway. The regulation of SeP might be a novel mechanism mediating the anti-diabetic effects of salsalate and adiponectin.

## Introduction

The liver is a pivotal organ in the regulation of glucose homeostasis and may modulate insulin resistance (IR) via the production of secreted proteins termed hepatokines [1]. Selenoprotein P (SeP) is a liver-derived protein, which was recently proposed to cause IR in liver and skeletal muscle. Using serial analysis of gene expression (SAGE) and DNA chip methods, Misu et al. found that hepatic SeP mRNA expression correlated with IR in humans [1]. SeP administration aggravated IR and glucose metabolism in both hepatocytes and myocytes. Conversely, both genetic deletion and RNA interference-mediated knockdown of SeP in mice led to an improvement in systemic IR and glucose tolerance [1]. The metabolic effects of SeP were mediated by the inhibition of adenosine monophosphate-activated protein kinase (AMPK) [1]. Circulating SeP levels were positively correlated with fasting plasma glucose and negatively associated with adiponectin in patients with type 2 diabetes mellitus (T2DM) [2]. In our recent study, serum SeP concentrations were significantly higher in patients with T2DM or prediabetes compared to those with normal glucose tolerance [3]. Furthermore, circulating SeP levels were associated with various cardiometabolic parameters including IR, inflammation, and atherosclerosis [3].

AMPK is a principal regulator of energy metabolism homeostasis, and AMPK signaling can inhibit inflammatory responses induced by the nuclear factor-κB (NF-κB) pathway [4]. Recently, Hawley et al. reported that salicylate directly activates AMPK [5]. Furthermore, in AMPK knockout mice, the effects of salicylate in increasing fat utilization and lowering plasma fatty acids disappeared [5]. Previous studies have shown that salicylate reverses hyperglycemia, hyperinsulinemia, and dyslipidemia [6]; however, side effects such as the risk of bleeding and gastric irritation limit its clinical utility. Salsalate is a prodrug of salicylate that is well tolerated and considered relatively safe for long-term clinical use [7]. In a recent multi-center randomized controlled trial, salsalate lowered HbA1c and triglyceride levels and increased adiponectin concentrations [8]. Previous studies have reported that adiponectin ameliorates hepatic IR and inflammation [9], [10]. Yamauchi et al. reported that adiponectin stimulates glucose utilization and fatty-acid oxidation by activating AMPK [11]. However, to the best of our knowledge, no previous reports have explored the effects of salsalate and adiponectin on IR via SeP modulation or the corresponding regulatory mechanisms.

In the present study, we investigated 1) the importance of SeP modulation in palmitate-induced IR in HepG2 cells; 2) the effects of salsalate and salicylate on SeP expression along with its regulatory mechanisms including AMPK and FOXO1α; 3) the influence of full-length adiponectin (fAd) on SeP expression and IR in HepG2 cells under hyperlipidemic conditions; and 4) the effects of salsalate and salicylate on hepatic SeP mRNA and protein expression along with glucose intolerance and IR in animal models.

## Materials and Methods

### Ethics Statement

The animal study was reviewed and approved by Institutional Animal Care and Use Committee (IACUC; No. KUIACUC-2012-156) of the Korea University, Seoul, Korea. The procedures for all animal experiments were performed according to IACUC guidelines.

### Cell Culture and Reagents

The human (15 year old Caucasian American male) hepatoma HepG2 cell line (ATCC, Manassas, VA, USA) was cultured in Dulbecco’s modified Eagle’s medium (DMEM) (Invitrogen, Carlsbad, CA, USA) supplemented with 10% fetal bovine serum (Invitrogen), 100 units/ml penicillin, and 100 µg/ml streptomycin (Invitrogen). Cells were incubated in a humidified atmosphere of 5% CO_2_ at 37°C. The HepG2 cells were cultured for 4 days to achieve 85% confluence before treatment with palmitate or other additives. Salsalate (Sigma, St. Louis, MO, USA) was dissolved in dimethyl sulfoxide (DMSO). Sodium salicylate (Sigma) was disolved in distilled water. Compound C (Sigma) and 5-aminoimidazole-4-carboxamide-1-β-d-ribonucleoside (AICAR; Sigma) were dissolved in DMSO and added to the culture medium. The final concentration of DMSO did not exceed 0.1%, which did not affect cell viability or AMPK phosphorylation. Sodium salt of palmitate (Sigma) was conjugated to 2% BSA (fatty acid-free; Sigma) and dissolved in DMEM in order to mimic the physiological concentration of albumin in human blood. In all experiments, cells were treated with palmitate-BSA for 24 hrs and 2% BSA was used as a control.

### Animals, Feeding, Treatment, and Glucose and Insulin Tolerance Tests

Six-week-old male Sprague–Dawley (SD) rats were conditioned at 24°C with a 12 h light/12 h dark cycle and fed a standard diet *ad libitum*. Rats were allowed to adapt to these conditions for 1 week before beginning the experimental protocol. The normal fat control group (n = 7) was reared on a normal fat diet (NFD, 10% energy from fat, D12450B, Research Diet Inc., USA) *ad libitum* for 14 weeks. The high fat control group (n = 7) was reared on a high fat diet (HFD, 60% energy from fat, D12492, Research Diet Inc.) *ad libitum* for 14 weeks. Rats in the salsalate treatment group (n = 7) were reared on a HFD for 8 weeks and received 200 mg/kg/day salsalate mixed into the HFD provided *ad libitum* for an additional 6 weeks. The intra-peritoneal glucose tolerance test (IPGTT) and insulin tolerance test (ITT) were performed according to protocols from a previous study [12]. Male homozygous B6.Cg-m +/+ Leprdb/J (*db/db*) mice at 5-week-old were given a NFD until 8-week-old. Wild-type C57BL/6J (B6) mice were used as lean control. *db/db* mice were injected IP once daily with 50 mg/kg/d salicylate for 5 weeks.

### RNA Extraction and Quantitative Real-time PCR

RNA from frozen liver tissues was isolated with Trizol (Invitrogen), and cDNA was synthesized with Superscript III (Invitrogen). Each cDNA sample was analyzed for gene expression by quantitative real-time PCR using the fluorescent TaqMan 5′-nuclease assay on an Applied Biosystems 7000 sequence detection system. TaqMan real-time PCR was performed using 2×TaqMan Master Mix and 20× premade TaqMan gene expression assays (Applied Biosystems, Foster City, CA, USA). The following PCR conditions were used: 45 cycles of 95°C for 10 minutes, 95°C for 15 seconds, and 60°C for 1 minute. The levels of rat SeP mRNA expression (Rn 00569905_ml; Applied Biosystems) was normalized to that of rat beta-actin (Rn 00667869_ml; Applied Biosystems). The levels of mouse SeP mRNA expression (Rn 00569905_ml; Applied Biosystems) was normalized to that of mouse beta-actin (Rn 00667869_ml; Applied Biosystems).

### Western Blot Analysis

HepG2 cells were harvested and extracted with lysis buffer (PRO-PREP™; Intron Biotechnology, Seoul, Korea) for 60 min at 4°C. Nuclear protein extracts were prepared using a protein fractionation kit (Biovision, Mountain View, CA, USA) according to the manufacturer’s directions. Protein samples (35 µg) were subjected to 10% SDS-PAGE, transferred to a nitrocellulose membrane (Amersham Bioscience, Westborough, MA, USA), and probed with primary antibody followed by secondary antibody conjugated with horseradish peroxidase (Amersham Bioscience). Anti-phospho IRS-1, anti-IRS-1, anti-phospho Akt, anti-Akt, anti-phospho AMPK, anti-AMPK, anti-phospho FOXO1α, and anti- FOXO1α were purchased from Cell Signaling (Beverly, MA, USA). Anti-SeP was purchased from Santa Cruz Biotechnology (Santa Cruz, CA, USA). The samples were detected with chemiluminescence kits (Amersham Bioscience).

### siRNA Transient Transfection

siRNA oligonucleotides for AMPK and SeP were purchased from Santa Cruz Biotechnology (Santa Cruz, CA, USA). A scrambled siRNA was used as a control. Transfection was performed with Lipofectamine 2000 (Invitrogen) per the manufacturer’s directions. In brief, siRNA or plasmid DNA was mixed with Lipofectamine 2000 in serum-free media. The cells were diluted in complete medium without antibiotics, resulting in 50–60% confluence 24 hrs after plating, after which they were mixed with siRNA or plasmid-liposome complexes. Samples were prepared 48 hrs after transfection.

### EMSA (Electrophoretic Mobility-shift Assay)

EMSA was performed using an EMSA kit (Panomics, Redwood City, CA, USA) according to the manufacturer’s directions. Nuclear extracts prepared from several treatment groups with oligonucleotide probes specific for the FOXO1α binding site (5′-TGAGGGGTGAGGTAAACAACAGGACTATAA-3′) in the SeP promoter region were used. For the supershift assay, 2 µg of anti-FOXO1α (Cell Signaling) was added to the nuclear extract, and the reaction mixture was resolved on a 6% (w/v) non-denaturing poly acrylamide gel. The samples were detected using a chemiluminescence kit (Amersham Bioscience).

### ChIP (Chromatin Immunoprecipitation) Assay

The ChIP assay was performed using the ChIP assay kit (Abcam, Cambridge, MA, USA) according to the manufacturer’s directions. Briefly, HepG2 cells were fixed, and chromatin was sheared by sonication. Chromatin complexes were immunoprecipitated for 12 hrs at 4°C using 7 µg of anti-FOXO1α antibody (Cell Signaling) or normal rabbit serum as a control. Immune complexes were harvested with 50 µl of protein A-agarose. Immunoprecipitated promoter fragments (PCR products) were visualized on a 1.5% agarose gel stained with ethidium bromide. The primers used for the amplification of human SeP promoter sequences were 5′- GCAAGGTCACTGCAAGAATGA-3′ (forward) and 5′-AAAGCCACAGCAGCACACTC-3′ (reverse).

### Statistical Analysis

All analyses were performed using the SPSS/PC statistical program (version 12.0 for Windows; SPSS, Inc., Chicago, IL, USA). Results are presented as the fold difference compared to control values (mean ± SE). All *in vitro* experiments were conducted a minimum of three times. Student’s t test or two-way ANOVA was used for statistical analysis.

## Results

### SeP is Involved in Palmitate-induced IR in HepG2 Cells

**Figure 1:**
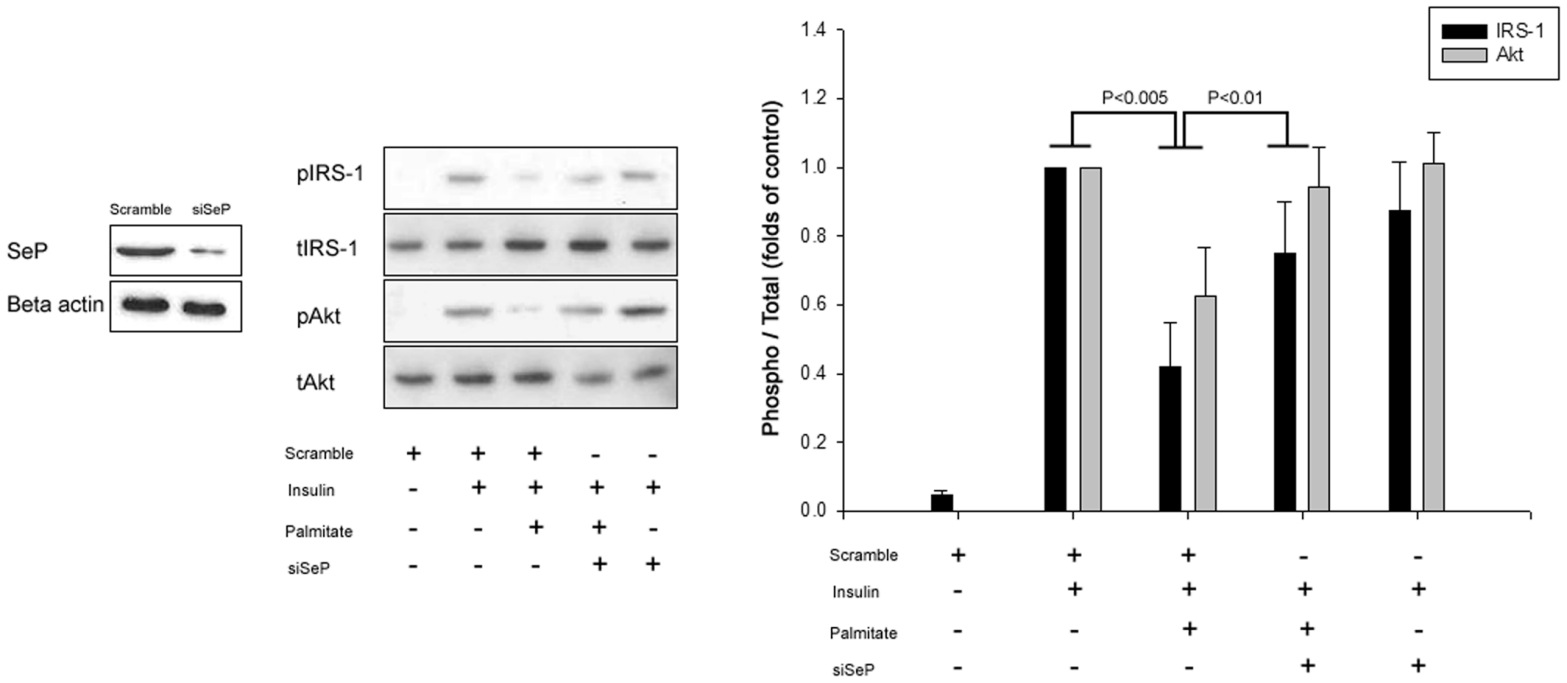
Palmitate significantly impairs insulin signaling, and selenoprotein P knock-down restores these changes in HepG2 cells. Control (scramble siRNA) or SeP siRNA-transfected HepG2 cells were incubated with 250 µM palmitate for 24 hr. The phosphorylation of IRS-1 (Tyr) and Akt (Ser) was determined by Western blot analysis. Insulin (10 nM) was used to stimulate IRS-1 and Akt for 3 min. Means ± SEMs were calculated from the results of three independent experiments.

We evaluated the role of SeP in insulin signaling in hepatocytes. Palmitate significantly inhibited insulin-stimulated IRS-1 (Tyr) and Akt (Ser) phosphorylation (**Figure 1**). However, with palmitate, the suppression of SeP expression by siRNA improved insulin signaling in HepG2 cells (**Figure 1**).

### Both Salsalate and Salicylate Inhibit Palmitate-induced SeP in HepG2 Cells

**Figure 2:**
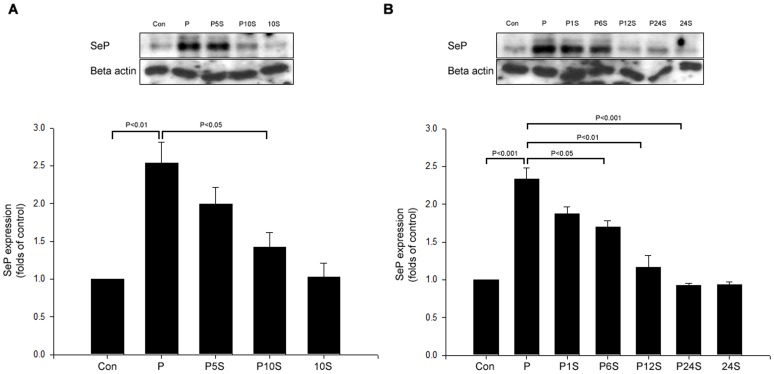
Salsalate inhibits palmitate-induced selenoprotein P expression in both dose- and time-dependent manners in HepG2 cells. (A) HepG2 cells were incubated with 250 µM palmitate (P) and different concentrations (mM) of salsalate for 24 hrs. After incubation, cell extracts were harvested and subjected to Western blot analysis to determine SeP expression. β-actin was used as an internal standard. (B) HepG2 cells were incubated with 250 µM palmitate (P) and 10 mM salsalate (S) for different periods (hr). After incubation, cell extracts were harvested and subjected to Western blot analysis to determine SeP levels. β-actin was used as an internal standard. Means ± SEMs were calculated from the results of three independent experiments.

We examined whether salsalate could inhibit palmitate-induced SeP expression in HepG2 cells. Palmitate significantly augmented SeP expression (**Figure 2**). Palmitate-induced SeP expression was decreased by salsalate treatment in both dose- and time-dependent manners (**Figure 2**). Salicylate, the primary metabolite of salsalate showed similar effects of salsalate on palmitate-induced SeP expression in HepG2 cells (**Figure S1**).

### Palmitate-induced SeP is Inhibited by both Salsalate and Salicylate through AMPK Activation

**Figure 3:**
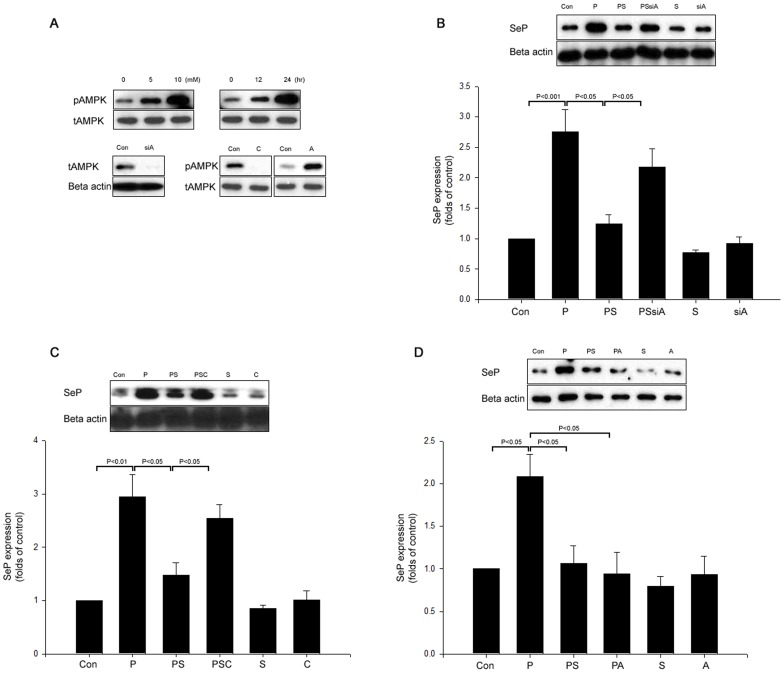
AMPK involves in the inhibitory effect of salsalate on palmitate-induced selenoprotein P in HepG2 cells. (A) HepG2 cells were incubated with different concentrations of salsalate for 24 hr or salsalate (10 mM) for different time periods. 20 µM compound C (C), AMPK siRNA (siA), and 2 mM AICAR (A) were tested. (B) Control (scramble siRNA) or AMPK siRNA (siA)-transfected HepG2 cells were incubated with 250 µM palmitate (P) and 10 mM salsalate (S) for 24 hr, and SeP expression was determined by Western blot analysis. (C) Control or 20 µM compound C (C)-treated HepG2 cells were incubated with 250 µM palmitate (P) and 10 mM salsalate (S) for 24 hr, and SeP expression was determined by Western blot analysis. (D) Control or AICAR (A)-treated HepG2 cells were incubated with 250 µM palmitate (P) and 10 mM salsalate (S) for 24 hr, and SeP expression was determined by Western blot analysis. β-actin was used as an internal standard. Means ± SEMs were calculated from the results of three independent experiments.

We verified that salsalate was capable of inducing AMPK phosphorylation in both dose- and time-dependent manners (**Figure 3A**). Furthermore, inhibition of palmitate-induced SeP by salsalate was markedly prevented by AMPK siRNA or an inhibitor of AMPK, such as compound C (**Figure 3B–C**). Conversely, AICAR, an activator of AMPK inhibited palmitate-induced SeP expression, similar to the effects of salsalate treatment (**Figure 3D**). Salicylate also inhibited palmitate-induced SeP through AMPK (**Figure S2**).

### Both Salsalate and Salicylate Suppress Palmitate-induced SeP through AMPK-mediated Phosphorylation of FOXO1α

**Figure 4:**
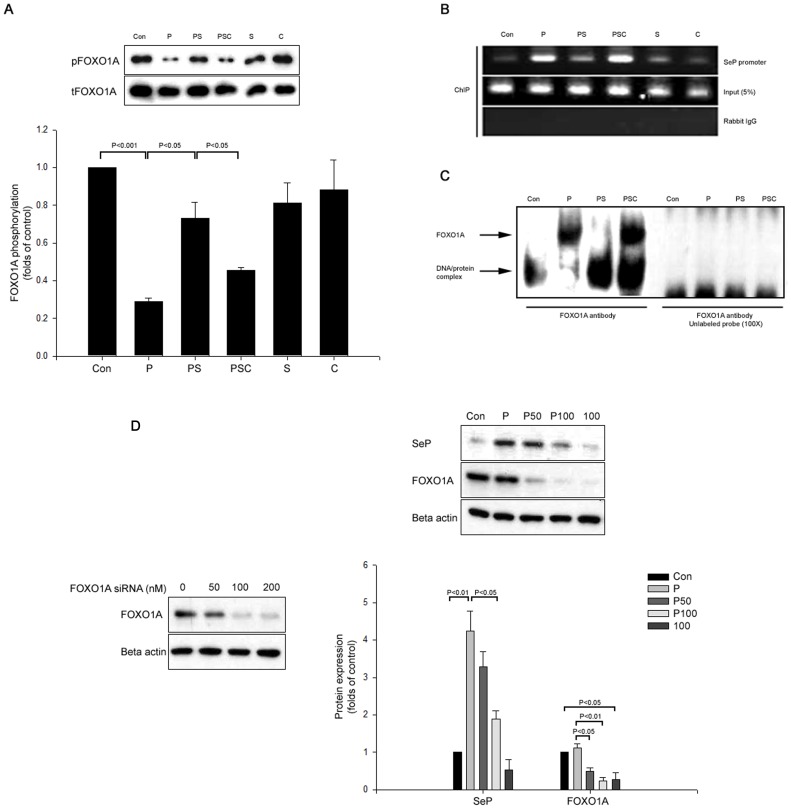
The inhibitory effect of salsalate on palmitate-induced selenoprotein P is involved in the AMPK-dependent FOXO1α pathway. (A) HepG2 cells were incubated with 250 µM palmitate (P) and 10 mM salsalate (S) or without salsalate or 20 µM compound C (C) for 24 hrs. FOXO1α phosphorylation (Ser) was determined by Western blot analysis with anti-FOXO1α and anti-phospho FOXO1α. (B) FOXO1α binding to the SeP promoter was determined using a ChIP assay. (C) Nuclear extracts from the above mentioned incubated cells were subjected to EMSA. For the supershift assay, an anti-FOXO1α antibody was used. An unlabeled probe was used to assess the specific binding of FOXO1α to the SeP promoter. Means ± SEMs were calculated from the results of three independent experiments.

Next, we evaluated whether salsalate was capable of preventing palmitate-induced SeP in hepatocytes via the AMPK-dependent FOXO1α pathway. Consistent with previous reports, palmitate dephosphorylated FOXO1α. However, salsalate reversed the palmitate-induced dephosphorylation of FOXO1α, and the effect of salsalate on FOXO1α dephosphorylation induced by palmitate was significantly inhibited by compound C (**Figure 4A**). Since the SeP promoter contains a FOXO1α response element [13], we tested the effect of salsalate on the palmitate-induced DNA binding activity of FOXO1α in HepG2 cells. We performed a ChIP assay and found that the binding activity of FOXO1α to the SeP promoter was markedly elevated by palmitate and prevented by salsalate. This inhibitory effect of salsalate was blocked by compound C (**Figure 4B**). Nuclear extracts obtained from palmitate-treated HepG2 cells, with or without salsalate treatment, were subjected to an EMSA-supershift assay using FOXO1α antibody and an oligonucleotide harboring the FOXO1α response element in the SeP promoter region. FOXO1α DNA binding was markedly increased by treatment with palmitate. However, salsalate significantly reduced the DNA binding activity of FOXO1α in response to palmitate. In contrast, the inhibitory effect of salsalate on palmitate-induced FOXO1α DNA binding activity was reversed by compound C (**Figure 4C**). Salicylate also showed inhibitory effects of salsalate on palmitate-induced SeP expression via FOXO1α modulation (**Figure S3**).

### Full-length Adiponectin Protects Against Palmitate-induced IR through Inhibition of SeP in HepG2 Cells

**Figure 5:**
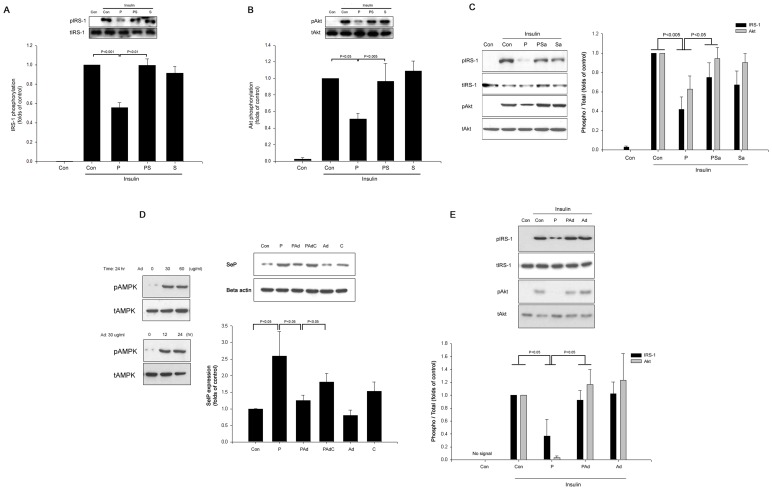
Salsalate, salicylate, and full-length adiponectin attenuate palmitate-induced insulin resistance in HepG2 cells. (A and B) HepG2 cells were incubated with 250 µM palmitate (P) and 10 mM salsalate (S) or without salsalate for 24 hrs. (C) HepG2 cells were incubated with 250 µM palmitate (P) and 10 mM salicylate (Sa) or without salicylate for 24 hrs. IRS-1 and Akt phosphorylation was measured by Western blot analysis. (D and E) HepG2 cells were incubated with different concentrations of fAd for 24 hr or fAd (30 µg/ml) for different time periods. HepG2 cells were incubated with 250 µM palmitate (P) and fAd (Ad) or without fAd for 24 hrs. AMPK, IRS-1, and Akt phosphorylations and SeP expression were determined by Western blot analysis. Insulin (10 nM) was used to stimulate IRS-1 and Akt for 3 min. Means ± SEMs were calculated from the results of three independent experiments.

We found that palmitate-induced IR in HepG2 cells was blocked by salsalate and salicylate (**Figure 5A–C**). Thus, we explored whether fAd treatment could suppress palmitate-induced SeP expression in HepG2 cells. We found that fAd treatment significantly prevented palmitate-induced SeP expression through the AMPK pathway and also improved insulin signaling in HepG2 cells (**Figure 5D–E**).

### Salsalate or Salicylate Treatments Inhibit SeP Expression and Ameliorate HFD- or Spontaneously-induced IR in Animal Models

**Figure 6:**
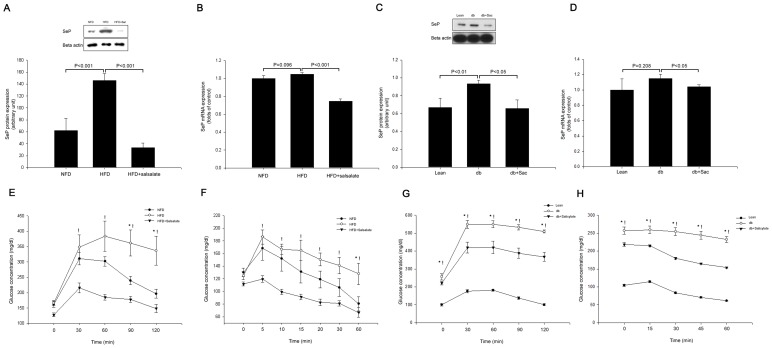
Systemic salsalate or salicylate treatments prevent high fat diet- or spontaneously-induced insulin resistance as well as hepatic selenoprotein P expression. (A) SeP protein expression was determined by Western blot analysis, (B) SeP mRNA expression was determined by real-time PCR analysis in normal fat diet (NFD), HFD, and HFD plus salsalate (HFD+Sal) treated SD rats (n = 7 animals per treatment group). (C) SeP protein expression was determined by Western blot analysis, (D) SeP mRNA expression was determined by real-time PCR analysis in B6 mice (Lean), *db/db* mice (db), and *db/db* mice plus salicylate (db+Sac) (n = 7 animals per treatment group). (E) Intra-peritoneal glucose tolerance test (IPGTT), (F) Insulin tolerance test (ITT) in normal fat diet (NFD,•), HFD (○), and HFD plus salsalate (HFD+Sal, ▾) treated SD rats (n = 7 animals per treatment group). (G) Intra-peritoneal glucose tolerance test (IPGTT), (H) Insulin tolerance test (ITT) in B6 mice (Lean, •), *db/db* mice (db, ○), and *db/db* mice plus salicylate (db+Sac, ▾) (n = 7 animals per treatment group). Means ± SEMs were calculated from the data for seven separate animals.

HFD-induced SeP mRNA and protein expression levels were significantly inhibited by salsalate treatment (**Figure 6A–B**). Salicylate also showed the inhibitory effects on both mRNA and protein expressions of SeP in *db/db* mice (**Figure 6C–D**). We next examined the effects of salsalate and salicylate on glucose tolerance and insulin sensitivity by performing an intra-peritoneal glucose tolerance test (IPGTT) and insulin tolerance test (ITT) in animal models as preliminary *in vivo* experiments. The IPGTT and ITT revealed that the HFD group and *db/db* mice had significantly impaired glucose tolerance and higher IR compared with the control group respectively. Furthermore, both SeP mRNA and protein expression levels in the liver were induced in HFD fed SD rats and *db/db* mice. However, salsalate or salicylate administrations significantly improved HFD- or spontaneously-induced glucose intolerance and IR (**Figure 6E–H**).

## Discussion

Recently, hepatokines such as fibroblast growth factor 21, fetuin-A, and SeP, have been proposed as potential targets for the treatment of T2DM [14], [15]. Misu et al. identified SeP as a novel hepatokine that regulates glucose homeostasis by modulating the insulin sensitivity of peripheral tissues in rodents and humans [1]. Hepatic SeP mRNA and serum SeP levels were found to be elevated in rodent models of T2DM, including OLETF rats and KKAy mice [1]. Treatment of primary hepatocytes with SeP induced a reduction in insulin-stimulated phosphorylation of insulin receptor and Akt [1]. In the present study, we found that palmitate significantly upregulates SeP expression in HepG2 hepatocytes, resulting in IR while knock-down of SeP by siRNA reverses these changes. Moreover, HFD or spontaneous obesity significantly upregulated hepatic SeP expression in animal models, accompanied by exacerbation of glucose intolerance and IR.

Low-grade, chronic inflammation may be a common factor linking obesity to IR, T2DM, and cardiovascular disease, and may participate in the pathogenesis of these obesity-related metabolic disorders [7]. The use of high-dose salicylates in obese, insulin resistant Zucker fatty rats and *ob/ob* mice significantly lowered blood glucose concentrations, improved glucose tolerance, and increased insulin sensitivity [16]. Although high-dose aspirin also improves glucose levels and insulin sensitivity in obese patients with T2DM [6], the negative side effects of prolonged high-dose aspirin intake precludes the application in patients with T2DM. Recent clinical trials revealed that salsalate, a prodrug of salicylate with fewer side effects than aspirin or salicylate, significantly reduced blood glucose and triglyceride levels [17]. However, the mechanism of action underlying the anti-diabetic effects of salsalate has not been fully elucidated.

Hawley et al. lately demonstrated that salicylate directly activates AMPK at concentrations reached in the plasma after the administration of salsalate or high-dose aspirin [5]. AMPK is a serine/threonine kinase with a central role in sensing energy status at the cellular level [4]. HFD-fed mice develop IR associated with suppressed AMPK phosphorylation [8]. Activated AMPK enhances the uptake and oxidation of glucose and fatty acids and induces mitochondrial biogenesis. AMPK has been suggested as an ideal drug target for the treatment of IR and T2DM [18]; anti-diabetic drugs such as metformin and thiazolidinediones work in part by activating AMPK [18]. AMPK has been reported to inhibit FOXO1α activity in hepatocytes by direct phosphorylation [19]. FOXO1α is a forkhead transcriptional factor that associates with insulin signaling on target gene expression in peripheral cells [20]. Activated FOXO1α boosts hepatic glucose production by inducing the gluconeogenic enzymes, phosphoenolpyruvate carboxykinase and glucose 6-phosphatase [21]. Abnormally increased FOXO1α activity, leading to insulin signaling impairment, is associated with pathogenesis in T2DM [22]. Palmitate has been reported to impair FOXO1α phosphorylation in cultured macrophages and hepatocytes [22], [23]. Recently, Lesniewski et al. showed that salicylate treatment improves age-associated vascular endothelial dysfunction through restoration of NFκB activation and FOXO3a phosphorylation [24]. Thus, we explored the effects of salsalate on SeP expression in palmitate-treated hepatocytes in association with the AMPK-FOXO1α pathway. We found for the first time that salsalate inhibited palmitate-induced SeP expression in both dose- and time-dependent manners. Furthermore, AMPK siRNA or compound C prevented these inhibitory effects of salsalate, whereas the effects of AICAR were similar to those of salsalate. In addition, palmitate-induced FOXO1α dephosphorylation and its binding to the SeP promoter were reversed by salsalate. These results suggest that salsalate may be a potential treatment strategy for IR and T2DM based on the mechanism of SeP inhibition through the AMPK-FOXO1α pathway.

Adiponectin is an adipose-derived hormone with a variety of beneficial functions including insulin sensitizing and anti-inflammatory properties. An et al. reported that adiponectin mRNA expression decreases in 3T3-L1 cells when they are treated with RAW 264.7-conditioned cell culture medium and that salsalate significantly reverses these changes [25]. Human trials showed that salsalate treatment markedly increases blood adiponectin concentrations and improves glucose and lipid metabolism [19]. Recently, Misu et al. compared serum levels of SeP with those of adiponectin in 36 patients with T2DM [2]. Circulating SeP levels were negatively associated with adiponectin levels. Furthermore, SeP knock-out mice exhibited an increase in blood adiponectin concentrations [2]. In this study, we found that fAd treatment inhibited palmitate-induced SeP expression through the activation of AMPK, accompanied by the attenuation of IR in hepatocytes. These findings suggest that SeP and adiponectin, which regulate IR in opposite directions, may mediate the coordination of metabolic control between the liver and adipose tissue.

Based on our findings from *in vitro* experiments, we further investigated whether salsalate and salicylate administrations may inhibit SeP mRNA and protein expression and improve glucose tolerance and insulin sensitivity. Indeed, the data from our preliminary *in vivo* experiment suggest that HFD- or spontaneously-induced IR and SeP expression are attenuated by salsalate or salicylate administrations respectively, which is consistent with the results from our *in vitro* experiments. We are now preparing for further detailed *in vivo* animal studies and human clinical trials to reinforce our current findings.

**Figure 7:**
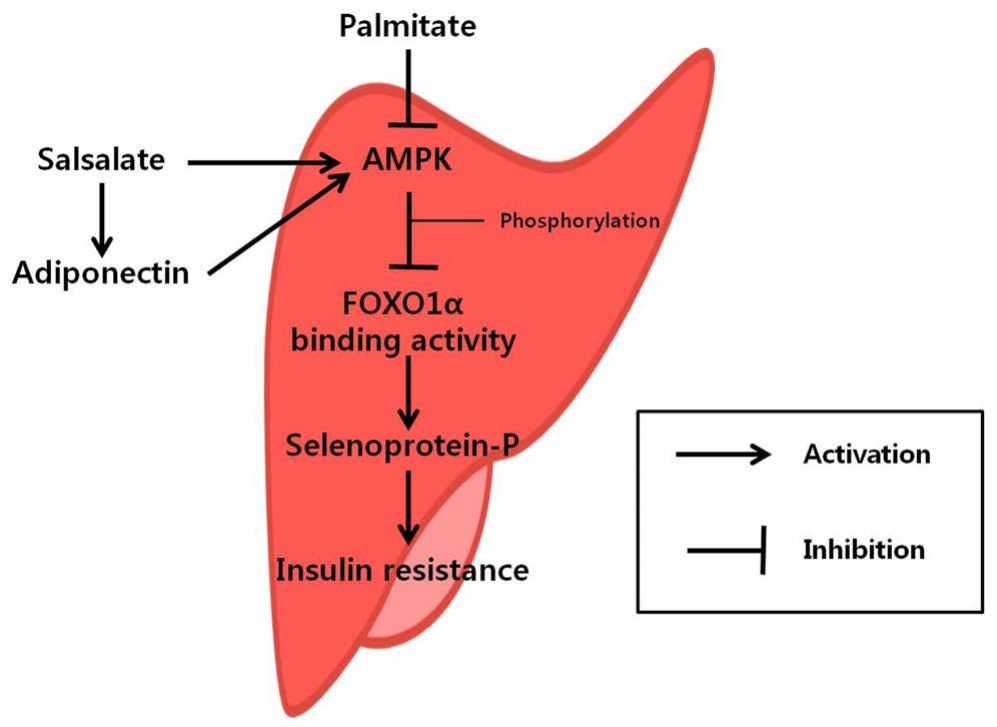
Schematic diagram illustrating the mechanism underlying the effect of salsalate and adiponectin on palmitate-induced insulin resistance.

In conclusion, salsalate and fAd inhibited SeP expression through the AMPK-FOXO1α-dependent pathway and consequently ameliorated palmitate-induced IR in hepatocytes (**Figure 7**). These results suggest that the regulation of SeP via the AMPK-FOXO1α-dependent pathway might be a novel mechanism mediating the anti-diabetic effects of salsalate and adiponectin.

## Supporting Information

Figure S1 **Salicylate inhibits palmitate-induced selenoprotein P expression in both dose- and time-dependent manners in HepG2 cells.** (A) HepG2 cells were incubated with 250 µM palmitate (P) and different concentrations (mM) of salicylate for 24 hrs. After incubation, cell extracts were harvested and subjected to Western blot analysis to determine SeP expression. β-actin was used as an internal standard. (B) HepG2 cells were incubated with 250 µM palmitate (P) and 10 mM salicylate (S) for different periods (hr). After incubation, cell extracts were harvested and subjected to Western blot analysis to determine SeP levels. β-actin was used as an internal standard. Means ± SEMs were calculated from the results of three independent experiments.
(TIF)

Figure S2 **AMPK involves in the inhibitory effect of salicylate on palmitate-induced selenoprotein P in HepG2 cells.** (A) HepG2 cells were incubated with different concentrations of salicylate for 24 hr or salicylate (10 mM) for different time periods. 20 µM compound C (C), AMPK siRNA (siAMPK), and 2 mM AICAR (A) were tested. (B) Control (scramble siRNA) or AMPK siRNA (siAMPK)-transfected HepG2 cells were incubated with 250 µM palmitate (P) and 10 mM salicylate (S) for 24 hr, and SeP expression was determined by Western blot analysis. (C) Control or 20 µM compound C (C)-treated HepG2 cells were incubated with 250 µM palmitate (P) and 10 mM salicylate (S) for 24 hr, and SeP expression was determined by Western blot analysis. (D) Control or AICAR (A)-treated HepG2 cells were incubated with 250 µM palmitate (P) and 10 mM salicylate (S) for 24 hr, and SeP expression was determined by Western blot analysis. β-actin was used as an internal standard. Means ± SEMs were calculated from the results of three independent experiments.
(TIF)

Figure S3 **The inhibitory effect of salicylate on palmitate-induced selenoprotein P is involved in the AMPK-dependent FOXO1α pathway.** (A) HepG2 cells were incubated with 250 µM palmitate (P) and 10 mM salicylate (S) or without salicylate or 20 µM compound C (C) for 24 hrs. FOXO1α phosphorylation was determined by Western blot analysis with anti-FOXO1α, anti-phospho FOXO1α, and anti- β-actin. β-actin was used as an internal standard. (B) FOXO1α binding to the SeP promoter was determined using a ChIP assay. (C) Nuclear extracts from the above mentioned incubated cells were subjected to EMSA. For the supershift assay, an anti-FOXO1α antibody was used. An unlabeled probe was used to assess the specific binding of FOXO1α to the SeP promoter. Means ± SEMs were calculated from the results of three independent experiments.
(TIF)
