# Supplementary figures and images for: Salsalate and Adiponectin Improve Palmitate-Induced Insulin Resistance via Inhibition of Selenoprotein P through the AMPK-FOXO1α Pathway
Source: PLoS One. 2013 Jun 18;8(6):e66529. doi: 10.1371/journal.pone.0066529 (PMC3689003; doi:10.1371/journal.pone.0066529)

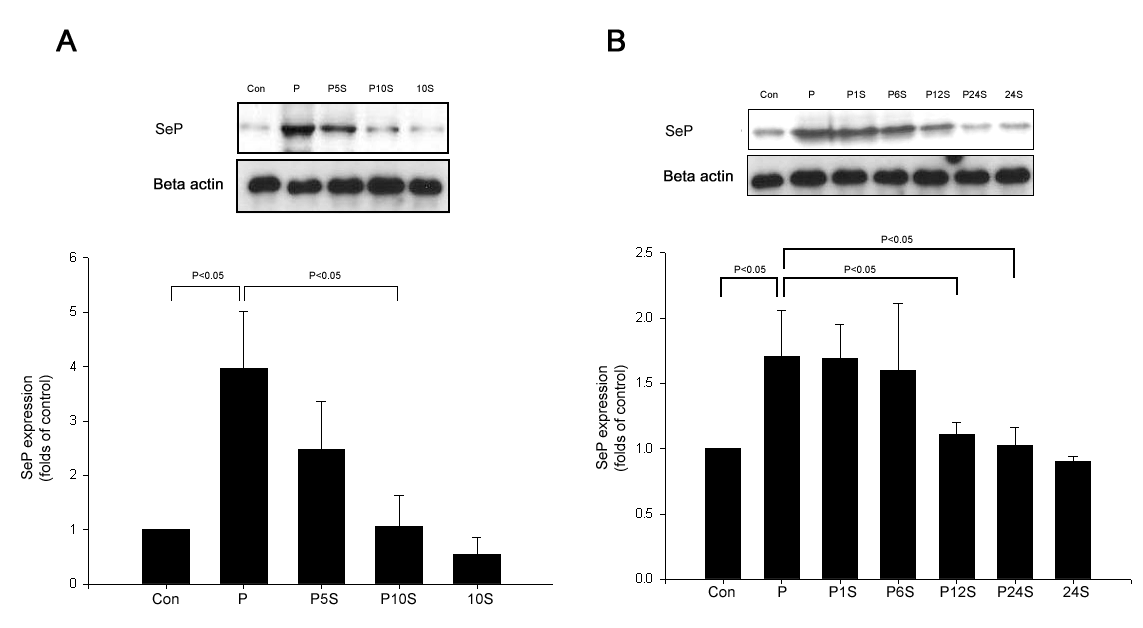

Supplement: Figure S1 — Salicylate inhibits palmitate-induced selenoprotein P expression in both dose- and time-dependent manners in HepG2 cells. (A) HepG2 cells were incubated with 250 µM palmitate (P) and different concentrations (mM) of salicylate for 24 hrs. After incubation, cell extracts were harvested and subjected to Western blot analysis to determine SeP expression. β-actin was used as an internal standard. (B) HepG2 cells were incubated with 250 µM palmitate (P) and 10 mM salicylate (S) for different periods (hr). After incubation, cell extracts were harvested and subjected to Western blot analysis to determine SeP levels. β-actin was used as an internal standard. Means ± SEMs were calculated from the results of three independent experiments. (TIF) [file pone.0066529.s001.tif]

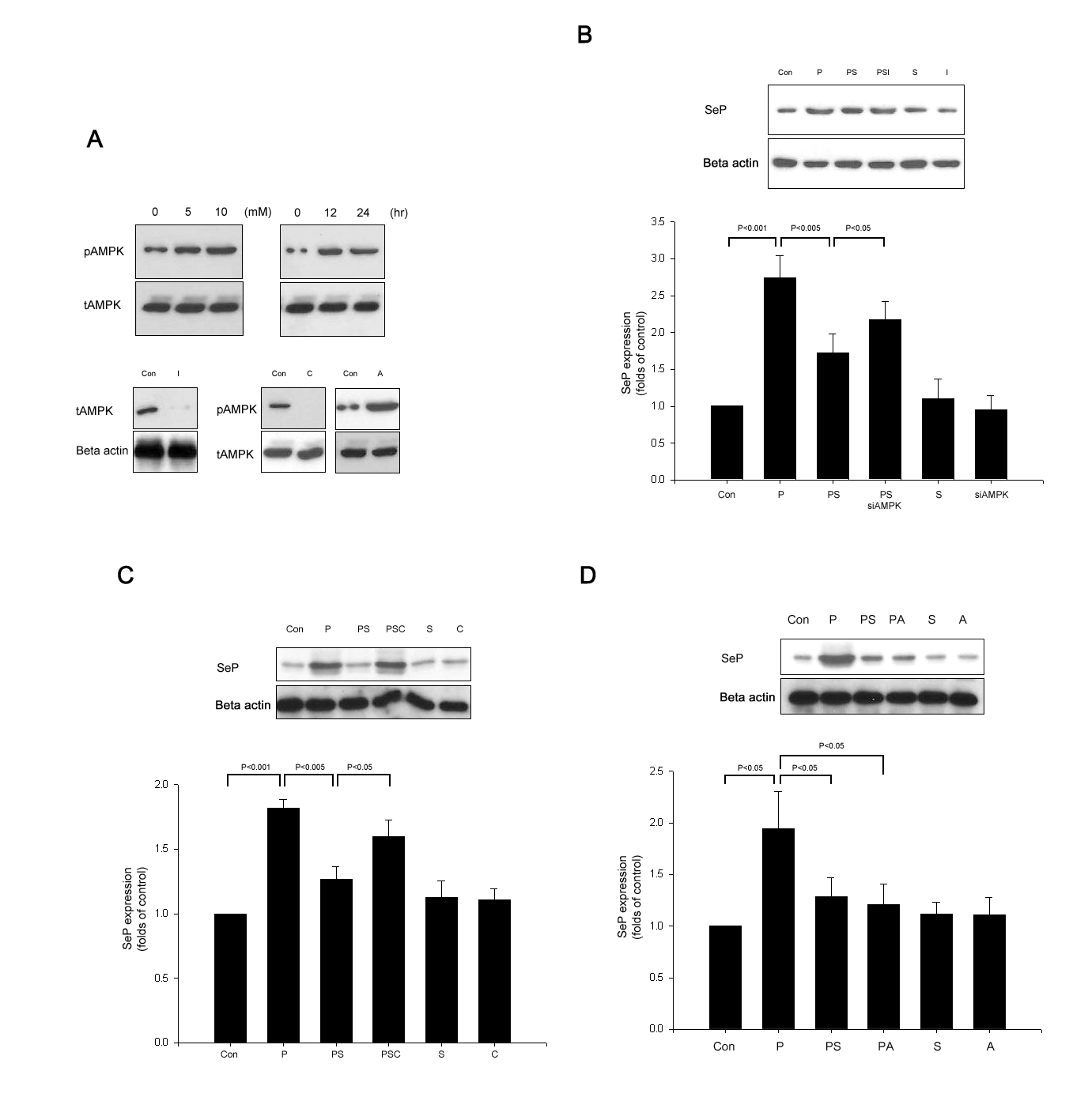

Supplement: Figure S2 — AMPK involves in the inhibitory effect of salicylate on palmitate-induced selenoprotein P in HepG2 cells. (A) HepG2 cells were incubated with different concentrations of salicylate for 24 hr or salicylate (10 mM) for different time periods. 20 µM compound C (C), AMPK siRNA (siAMPK), and 2 mM AICAR (A) were tested. (B) Control (scramble siRNA) or AMPK siRNA (siAMPK)-transfected HepG2 cells were incubated with 250 µM palmitate (P) and 10 mM salicylate (S) for 24 hr, and SeP expression was determined by Western blot analysis. (C) Control or 20 µM compound C (C)-treated HepG2 cells were incubated with 250 µM palmitate (P) and 10 mM salicylate (S) for 24 hr, and SeP expression was determined by Western blot analysis. (D) Control or AICAR (A)-treated HepG2 cells were incubated with 250 µM palmitate (P) and 10 mM salicylate (S) for 24 hr, and SeP expression was determined by Western blot analysis. β-actin was used as an internal standard. Means ± SEMs were calculated from the results of three independent experiments. (TIF) [file pone.0066529.s002.tif]

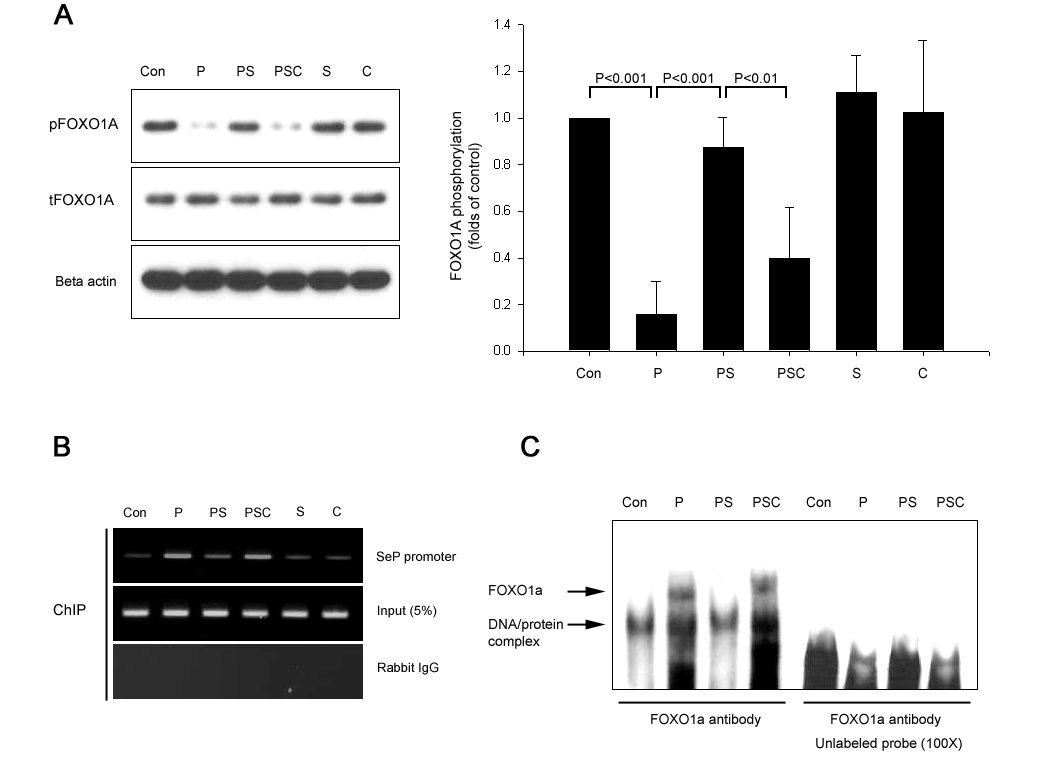

Supplement: Figure S3 — The inhibitory effect of salicylate on palmitate-induced selenoprotein P is involved in the AMPK-dependent FOXO1α pathway. (A) HepG2 cells were incubated with 250 µM palmitate (P) and 10 mM salicylate (S) or without salicylate or 20 µM compound C (C) for 24 hrs. FOXO1α phosphorylation was determined by Western blot analysis with anti-FOXO1α, anti-phospho FOXO1α, and anti- β-actin. β-actin was used as an internal standard. (B) FOXO1α binding to the SeP promoter was determined using a ChIP assay. (C) Nuclear extracts from the above mentioned incubated cells were subjected to EMSA. For the supershift assay, an anti-FOXO1α antibody was used. An unlabeled probe was used to assess the specific binding of FOXO1α to the SeP promoter. Means ± SEMs were calculated from the results of three independent experiments. (TIF) [file pone.0066529.s003.tif]
